# Percutaneous coronary intervention improves quality of life of patients with chronic total occlusion and low estimated glomerular filtration rate

**DOI:** 10.3389/fcvm.2022.1019688

**Published:** 2022-12-21

**Authors:** Shuai Zhao, Yan Chen, Boda Zhu, Jiayi Wang, Zhihong Wei, Yiming Zou, Wentao Hu, Genrui Chen, Huan Wang, Chenhai Xia, Tiantong Yu, Peng Han, Li Yang, Wei Wang, Zhongjie Zhai, Haokao Gao, Chengxiang Li, Kun Lian

**Affiliations:** ^1^Department of Cardiology, Xijing Hospital, The Fourth Military Medical University, Xi'an, Shaanxi, China; ^2^Department of Cardiology, No. 971 Hospital of the PLA Navy, Qingdao, Shandong, China; ^3^Primary Flight Training Base, Air Force Aviation University, Harbin, Heilongjiang, China; ^4^Cadet Brigade, School of Basic Medicine, The Fourth Military Medical University, Xi'an, Shaanxi, China; ^5^Department of Cardiology, Hanyin County People's Hospital, Ankang, Shaanxi, China; ^6^Department of Cardiology, 981 Hospital of Joint Logistics Support Force, Chengde, Hebei, China; ^7^Department of Pharmaceutics and Pharmacy Administration, School of Pharmacy, The Fourth Military Medical University, Xi'an, Shaanxi, China; ^8^Department of Health Statistics, The Fourth Military Medical University, Xi'an, Shaanxi, China

**Keywords:** chronic total occlusion, percutaneous coronary intervention, low estimated glomerular filtration rate, symptom, quality of life

## Abstract

**Background:**

A low estimated glomerular filtration rate (eGFR <90 mL/min/1.73 m^2^) is widely recognized as a risk factor for major adverse cardiac events (MACE) after percutaneous coronary intervention (PCI) for chronic total occlusion (CTO). However, the impact of successful CTO-PCI on quality of life (QOL) of patients with low eGFR remains unknown.

**Objectives:**

The aim of this prospective study was to assess the QOL of CTO patients with low eGFR after successful PCI.

**Methods:**

Consecutive patients undergoing elective CTO-PCI were prospectively enrolled and subdivided into four groups: eGFR ≥90 mL/min/1.73 m^2^ (*n* = 410), 90 > eGFR ≥ 60 mL/min/1.73 m^2^ (*n* = 482), 60 > eGFR ≥ 30 mL/min/1.73 m^2^ (*n* = 161), and eGFR <30 mL/min/1.73 m^2^ (*n* = 23). The primary outcomes included QOL, as assessed with the European Quality of Life-5 Dimensions (EQ-5D) questionnaire, and symptoms, as assessed with the Rose Dyspnea Scale (RDS) and Seattle Angina Questionnaire (SAQ), at 1 month and 1 year after successful PCI.

**Results:**

With the decline of eGFR, CTO patients were more likely to present with comorbidities of hypertension, diabetes, hyperuricemia, and previous stroke, in addition to lower hemoglobin levels and left ventricular ejection fraction (*p* < 0.05). Low eGFR was associated with greater incidences of in-hospital pericardiocentesis, major bleeding, acute renal failure, and subcutaneous hematoma, but not in-hospital MACE (*p* < 0.05). Symptoms of dyspnea and angina were alleviated in all CTO patients with eGFR ≥30 mL/min/1.73 m^2^ at 1 month and 1 year after successful CTO-PCI, but only at 1 month for those with eGFR <30 mL/min/1.73 m^2^ (*p* < 0.01). Importantly, QOL was markedly improved at 1 month and 1 year after successful PCI (*p* < 0.01), notably at a similar degree between patients with low eGFR and those with normal eGFR (*p* > 0.05).

**Conclusion:**

Successful PCI effectively improved symptoms and QOL of CTO patients with low eGFR.

## Introduction

Chronic total occlusion (CTO) is reported in ~13–41% of patients with coronary artery disease (CAD) and represents one of the last barriers to percutaneous coronary intervention (PCI) due to lesion complexity, with lower procedural success rates and greater complication rates, radiation exposure, and procedural duration (1–4). However, with the recent development of interventional techniques and dedicated equipment, the success rate of CTO-PCI can reach 92% among experienced surgeons (5). More importantly, successful CTO-PCI can prolong long-term survival, relieve symptoms, and improve ventricular function (6–9). Among CTO patients, low estimated glomerular filtration rate (eGFR <90 mL/min/1.73 m^2^) is widely recognized as an important high-risk factor for in-hospital complications, major adverse cardiac events (MACE), and all-cause mortality after successful PCI (2, 7, 10–17). However, the primary goal of CTO-PCI is generally to improve symptoms and quality of life (QOL) (18). Previous studies have reported that low eGFR is associated with severe angina and reduced QOL (19, 20), thereby emphasizing the importance of the symptoms and QOL of CTO patients with low eGFR. A report by the OPEN-CTO registry (Outcomes, Patient Health Status, and Efficiency in Chronic Total Occlusion Hybrid Procedures) found that successful CTO-PCI significantly improved angina of patients with low eGFR (<60 mL/min/1.73 m^2^) (21), although there is no data to determine whether successful CTO-PCI will actually improve QOL of these patients. Therefore, to address this gap in knowledge, the aim of this prospective study was to assess the effect of successful CTO-PCI on symptoms of angina and dyspnea and QOL of patients with low eGFR to provide real-world experience for revascularization of CTO patients with low eGFR.

## Methods

### Patient population

**Figure 1:**
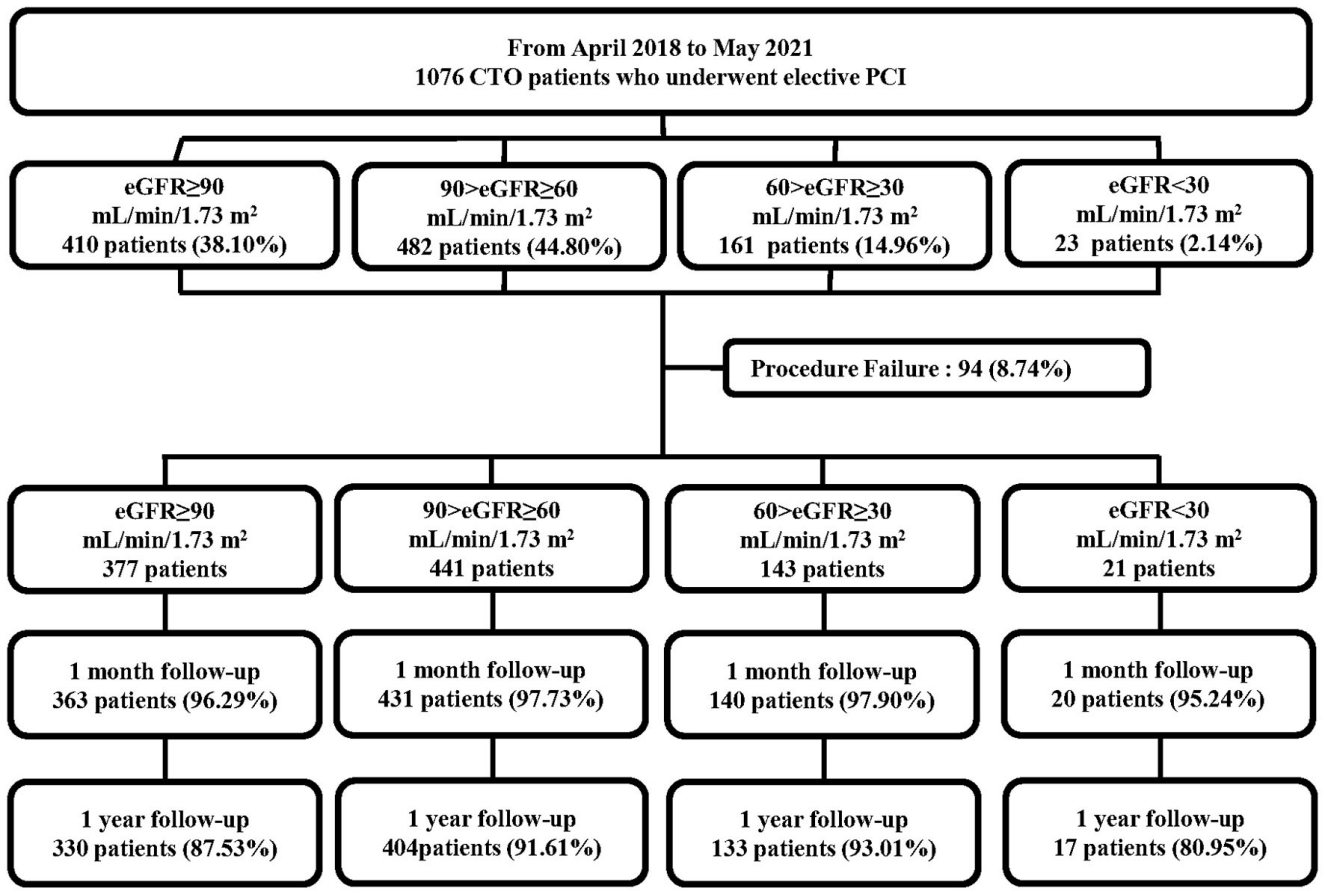
Flowchart of the study population. CTO, chronic total occlusion; PCI, percutaneous coronary intervention; eGFR, estimated glomerular filtration rate.

In total, 1,076 consecutive patients who underwent elective PCI for at least 1 CTO lesion in the period between April 2018 and May 2021 were prospectively enrolled in this study (Figure 1). All the procedures were performed at Xijing Hospital (Xi'an, Shaanxi, China) by one CTO team led by Dr. Chengxiang Li who personally completed or guided all procedures. Patients with acute myocardial infarctions (ST or non-ST elevation), cardiogenic shock, and unstable hemodynamics were excluded. Indication for coronary revascularization was based on angina symptoms or non-invasive imaging (computed tomography) of the coronary artery. The revascularization strategy (PCI or coronary-artery bypass grafting and lesions to be revascularized) was individualized for each patient and left to the discretion of the cardiac surgeon and interventionalists in our center. If the patient rejected the surgical recommendation, PCI was proposed if considered feasible by the chief surgeon.

The eGFR was calculated at baseline for each patient using the Modification in Diet in Renal Disease equation (22). Based on the eGFR, the study cohort was subdivided into four groups, consistent with prior publications (16, 17, 23): eGFR ≥90 mL/min/1.73 m^2^, 90 > eGFR ≥ 60 mL/min/1.73 m^2^, 60 > eGFR ≥ 30 mL/min/1.73 m^2^, and eGFR <30 mL/min/1.73 m^2^. The study protocol was approved by the Ethics Committee of Xijing Hospital (approval no. KY20172019-1) and each subject provided informed consent before recruitment.

### Definition and endpoints

Coronary CTO was defined as angiographic evidence of total occlusion with thrombolysis in myocardial infarction flow grade 0 within a major epicardial coronary artery of at least 2.5 mm, with an estimated duration of at least 3 months. Non-CTO was defined as diameter stenosis of 50% for left main (LM) or 70% for non-LM CAD within a vessel diameter ≥2.5 mm (23). Revascularization was considered for angiographically significant stenosis (≥70% diameter reduction by visual assessment) and functionally significant stenosis (fractional flow reserve measurement <0.80). Complete revascularization was defined as revascularization of all significantly diseased major epicardial vessels during the same hospitalization. The J-CTO score (Multicenter CTO Registry in Japan) was calculated as previously described (24). Procedural success was defined as successful CTO revascularization with achievement of <30% residual diameter stenosis within the treated segment and restoration of Thrombolysis in Myocardial Infarction flow grade 3 (antegrade) and no in-hospital MACE. In-hospital MACE included any of the following adverse events prior to hospital discharge: all-cause mortality, non-fatal myocardial infarction, and clinically driven revascularization. Major bleeding was defined as Bleeding Academic Research Consortium type 3 or greater (25).

### Follow-up

Patients were followed-up by a clinical visit or telephone interview at 1 month and 1 year after CTO-PCI. The outcomes of interest for this study were changes to symptoms, including dyspnea and angina, and QOL. Details regarding symptoms and QOL were obtained from hospital re-admission records and outpatient visits.

### Symptom assessment

Dyspnea was assessed at baseline and then at 1 month and 1 year after CTO-PCI in compliance with the Rose Dyspnea Scale (RDS). The RDS is a four-item questionnaire to assess the level of dyspnea while performing common activities (26), where each activity associated with dyspnea is assigned 1 point. RDS scores range from 0 to 4, with a score of 0 indicating no dyspnea and increased scores indicating greater severity of dyspnea.

The angina status of the patient was assessed with the Seattle Angina Questionnaire (SAQ) (27) at baseline and then at 1 month and 1 year after CTO-PCI. The SAQ consists of 19 items that measure five dimensions: angina frequency (AF), angina stability (AS), disease perception, physical limitation, and treatment satisfaction. All items were assessed with a five-point descriptive scale and the total score was calculated as the sum of all individual scores within each group and transformed to a scale of 0 to 100, where 0 is the worst and 100 is the best.

### QOL assessment

QOL was assessed with the use of the European Quality of Life-5 Dimensions (EQ-5D) questionnaire at baseline and then at 1 month and 1 year after CTO-PCI. The EQ-5D questionnaire assesses five dimensions of general health (mobility, self-care, usual activities, pain/discomfort, and anxiety/depression) with a three-point scale. The EQ-5D scores were converted to utilities with an algorithm developed for a Japanese population. Utilities are preference-weighted health status assessments with scores ranging from −0.11 to 1.00, with 1.00 representing perfect health and −0.11 representing poorest health (28, 29).

### Statistical analysis

Study participants were categorized according to the baseline eGFR. The baseline characteristics, angiographic characteristics and procedural details, intraprocedural and in-hospital complications were shown in Tables 1–**3**. Continuous variables are presented as the mean ± standard deviation or the median and interquartile range, whereas categorical variables are presented as percentages. Continuous variables were compared with the ANOVA test (normal distribution) or Kruskal–Wallis *H* test (abnormal distribution). Categorical variables were compared with the chi-square test or Kruskal–Wallis *H* test (NYHA functional class). The changes of symptoms and QOL (EQ-5D) were showed in **Tables 5**, **7**. The comparison between two groups was used the student *t*-test while the comparison among four groups was used ANOVA test. Univariable and multivariable binary logistic regression analysis were performed to identify the risk factors of in-hospital complications and 1-year symptoms and QOL improvement in patients with low eGFR (<90 mL/min/1.73 m^2^) after successful CTO-PCI (**Tables 4**, **6**, **8**). A two-sided probability (*p*) value of <0.05 was considered significant. All calculations were performed with IBM-SPSS Statistics for Windows, version 25.0 (IBM Corporation, Armonk, NY, USA) and STATA 14 software (https://www.stata.com/stata14/).

**Table 1:** Baseline characteristics.

	**eGFR ≥90** **mL/min/1.73 m^2^**	**90 > eGFR ≥60** **mL/min/1.73 m^2^**	**60 > eGFR ≥30** **mL/min/1.73 m^2^**	**eGFR < 30** **mL/min/1.73 m^2^**	***p*-value**
	**(*n* = 410)**	**(*n* = 482)**	**(*n* = 161)**	**(*n* = 23)**	
Age, yrs	57.53 ± 10.56	61.46 ± 10.29	65.88 ± 9.96	61.13 ± 9.97	< 0.001
Males, *n*%	374 (91.22)	422 (87.55)	128 (79.50)	18 (78.26)	0.001
BMI, kg/m^2^	25.25 ± 3.20	25.33 ± 3.17	24.91 ± 3.01	23.53 ± 3.33	0.035
SBP, mmHg	127.61 ± 18.53	126.80 ± 20.29	127.75 ± 20.88	135.96 ± 26.52	0.188
DBP, mmHg	73.47 ± 11.59	71.64 ± 12.29	70.57 ± 12.17	76.96 ± 15.86	0.008
Smoking, *n*%	183 (44.63)	171 (35.48)	42 (16.15)	4 (17.39)	< 0.001
Hypertension, *n*%	236 (57.56)	288 (59.75)	117 (72.67)	23 (100)	< 0.001
Diabetes, *n*%	138 (33.66)	147 (30.50)	79 (49.07)	10 (43.48)	< 0.001
Previous MI, *n*%	164 (40.00)	193 (40.04)	75 (46.58)	11 (47.83)	0.415
Previous PCI, %	208 (50.73)	235 (48.76)	91 (56.52)	12 (52.17)	0.402
Previous CABG, %	8 (1.95)	19 (3.94)	8 (4.97)	0 (0)	0.157
Previous stroke, *n*%	33 (8.05)	71 (14.73)	23 (14.29)	3 (13.04)	0.017
Chronic pulmonary disease, *n*%	7 (1.71)	9 (1.87)	6 (3.73)	0 (0)	0.382
Peripheral artery disease, *n*%	8 (1.95)	13 (2.70)	5 (3.11)	1 (4.35)	0.767
Family history of CHD, *n*%	17 (4.15)	18 (3.73)	6 (3.73)	3 (13.04)	0.178
WBC, *10^9^/L	7.02 ± 1.91	6.85 ± 2.13	7.09 ± 2.08	7.57 ± 2.44	0.230
Platelet, *10^9^/L	212.89 ± 65.54	204.05 ± 60.13	205.94 ± 76.89	211.65 ± 59.90	0.228
Hemoglobin, g/L	143.20 ± 16.98	140.12 ± 17.78	133.51 ± 23.59	109.09 ± 20.98	< 0.001
FBG, mmol/L	6.53 ± 2.78	6.31 ± 2.94	6.59 ± 2.56	6.49 ± 3.05	0.652
TC, mmol/L	3.42 ± 1.08	3.37 ± 1.00	3.33 ± 0.85	3.48 ± 0.92	0.699
TG, mmol/L	1.65 ± 1.20	1.66 ± 1.02	1.64 ± 0.88	2.07 ± 1.80	0.362
LDL-C, mmol/L	1.95 ± 0.94	1.87 ± 0.88	1.84 ± 0.77	1.90 ± 0.84	0.513
HDL-C, mmol/L	1.02 ± 0.41	1.00 ± 0.34	1.01 ± 0.51	1.00 ± 0.35	0.746
ALT, U/L	31.66 ± 27.80	32.62 ± 41.50	31.89 ± 64.41	18.76 ± 17.59	0.514
AST, U/L	26.16 ± 27.65	27.31 ± 26.56	32.30 ± 71.75	18.04 ± 10.67	0.195
Scr, μmol/L	66.24 ± 8.19	87.25 ± 10.84	123.38 ± 23.85	390.57 ± 250.96	< 0.001
eGFR, mL/min per 1.73 m^2^	106.82 ± 14.68	75.60 ± 8.53	49.71 ± 8.21	17.91 ± 8.61	< 0.001
CrCL, ml/min	111.52 ± 25.38	80.26 ± 18.85	52.92 ± 15.47	21.03 ± 10.95	< 0.001
Uric acid, μmol/L	319.07 ± 81.00	355.59 ± 91.93	405.48 ± 123.50	384.39 ± 143.59	< 0.001
Hyperuricemia, *n*%	41 (10.00)	97 (20.12)	58 (36.02)	12 (52.17)	< 0.001
cTnI, ng/mL	0.82 ± 8.74	0.65 ± 4.87	1.81 ± 12.24	0.09 ± 0.08	0.408
NT-proBNP, pg/ml	636.34 ± 1516.92	953.30 ± 2077.72	2358.72 ± 4561.90	13576.22 ± 13891.73	< 0.001
CK, IU/L	128.94 ± 319.17	125.28 ± 190.63	163.15 ± 426.96	162.94 ± 280.08	0.599
CK-MB, IU/L	17.80 ± 35.28	16.40 ± 21.64	17.83 ± 28.73	21.93 ± 22.74	0.821
LVEF, %	51.70 ± 8.55	50.27 ± 9.84	46.42 ± 11.32	46.35 ± 8.73	< 0.001
Dyspnea (NYHA functional class), *n*%					0.421
I	112 (27.32)	133 (27.59)	39 (24.22)	6 (26.08)	0.862
II	191 (46.59)	246 (51.04)	74 (45.96)	9 (39.13)	0.411
III	87 (21.22)	87 (18.05)	41 (25.47)	5 (21.74)	0.383
IV	20 (4.88)	16 (3.32)	7 (4.35)	3 (13.04)	0.224
NYHA functional class (III/IV), *n*%	107 (26.10)	103 (21.37)	48 (29.81)	8 (34.78)	0.078
Medications on discharge, *n*%					
Aspirin	393 (95.85)	449 (93.15)	153 (95.03)	15 (65.22)	< 0.001
P2Y12 inhibitors	408 (99.51)	479 (99.38)	161 (100.00)	23 (100.00)	0.772
Statin	405 (98.78)	476 (98.76)	159 (98.76)	22 (95.65)	0.637
ACE inhibitors or ARB	329 (80.24)	404 (83.82)	141 (87.58)	15 (65.22)	0.021
β-Blockers	361 (88.05)	449 (93.15)	144 (89.44)	23 (100.00)	0.022

eGFR, estimated glomerular filtration rate; BMI, body mass index; SBP, systolic blood pressure; DBP, diastolic blood pressure; MI, myocardial infarction; PCI, percutaneous coronary intervention; CABG, coronary artery bypass grafting; CHD, coronary atherosclerotic heart disease; WBC, white blood cell; FBG, fasting blood glucose; TC, total cholesterol; TG, triglyceride; LDL-C, low density lipoprotein cholesterol; HDL-C, high density lipoprotein cholesterol; ALT, alanine aminotransaminase; AST, aspartate aminotransferase; Scr, serum creatinine; CrCL, creatinine clearance; cTnI, cardiac troponin I; NT-proBNP, N-terminal pro-B type natriuretic peptide; CK, creatine kinase; CK-MB, creatine kinase MB; LVEF, left ventricular ejection fraction; NYHA, New York Heart Association; ACE, angiotensin-converting enzyme; ARB, angiotensin receptor blockers.

## Results

The 1,076 patients were subdivided into four groups according to baseline eGFR. A flowchart of the patient selection process is presented in Figure 1. Overall, lower eGFR tended to occur in older and female CTO patients and those with hypertension, diabetes, hyperuricemia, and previous stroke. Meanwhile, CTO patients with low eGFR generally had lower body mass index, hemoglobin level, creatinine clearance, and left ventricular ejection fraction (LVEF), and higher diastolic blood pressure, serum creatinine, uric acid, and N-terminal pro-brain natriuretic peptide (NT-proBNP) (*p* < 0.05, Table 1). Angiographic features and procedural details are shown in Table 2. Among the CTO patients, low eGFR was correlated with a greater number of lesions, CTO of the right coronary artery, and fewer “interventional” collaterals (*p* < 0.05).

**Table 2:** Angiographic characteristics and procedural details.

	**eGFR ≥90** **mL/min/1.73 m^2^**	**90 > eGFR ≥60** **mL/min/1.73 m^2^**	**60 > eGFR ≥30** **mL/min/1.73 m^2^**	**eGFR < 30** **mL/min/1.73 m^2^**	***p*-value**
	**(*n* = 410)**	**(*n* = 482)**	**(*n* = 161)**	**(*n* = 23)**	
Vascular lesion, *n*%					
LM lesion	67 (13.34)	106 (21.99)	35 (21.74)	3 (13.04)	0.132
LAD lesion	341 (83.17)	399 (82.78)	141 (87.57)	20 (86.96)	0.505
LCX lesion	293 (71.46)	348 (72.20)	128 (79.50)	17 (73.91)	0.249
RCA lesion	312 (76.10)	391 (81.12)	132 (81.99)	18 (78.26)	0.235
Multivessel disease, *n*%	351 (85.61)	413 (85.68)	148 (91.93)	19 (82.60)	0.178
Number of lesions per patient	2.38 ± 0.97	2.51 ± 0.97	2.63 ± 0.93	2.26 ± 1.14	0.017
Location of the CTO, *n*%					
LM-CTO	4 (0.98)	5 (1.04)	1 (0.62)	0 (0)	0.929
LAD-CTO	195 (47.56)	228 (47.30)	63 (39.13)	10 (43.48)	0.279
LCX-CTO	116 (28.29)	141 (29.25)	57 (35.40)	8 (34.78)	0.364
RCA-CTO	231 (56.34)	279 (57.88)	112 (69.57)	13 (56.52)	0.030
Multi-CTO lesion, *n*%	118 (28.78)	148 (30.71)	58 (36.02)	7 (30.43)	0.415
Number of CTO per patient	1.33 ± 0.55	1.35 ± 0.57	1.45 ± 0.65	1.35 ± 0.57	0.180
CTO target vessel, *n*%					
LM-CTO	2 (0.49)	5 (1.04)	1 (0.62)	0 (0)	0.769
LAD-CTO	162 (39.51)	194 (40.25)	47 (29.19)	10 (43.48)	0.074
LCX-CTO	63 (15.37)	70 (14.52)	24 (14.90)	2 (8.70)	0.846
RCA-CTO	200 (48.78)	231 (47.93)	96 (59.63)	12 (52.17)	0.069
Ostial location, *n*%	52 (12.68)	42 (8.71)	9 (5.59)	4 (17.39)	0.028
In-stent occlusion, *n*%	39 (9.51)	36 (7.47)	14 (8.70)	0 (0)	0.340
Lesion length, mm	27.91 ± 22.18	27.78 ± 16.81	28.75 ± 17.98	31.58 ± 26.81	0.816
Lesion length ≥20 mm, *n*%	277 (67.56)	315 (65.35)	101 (62.73)	12 (52.17)	0.372
Blunt stump, *n*%	277 (67.56)	335 (69.50)	106 (65.84)	15 (65.22)	0.811
Tortuosity ≥45°, *n*%	101 (24.63)	145 (30.08)	55 (34.16)	5 (21.74)	0.084
Calcification, *n*%	137 (33.41)	168 (34.85)	41 (25.47)	10 (43.48)	0.109
Reattempt, *n*%	75 (18.29)	68 (14.11)	24 (14.91)	3 (13.04)	0.365
J-CTO score	2.18 ± 1.14	2.19 ± 1.13	2.12 ± 1.16	2.37 ± 1.38	0.819
SYNTAX score	29.33 ± 11.65	30.68 ± 12.17	31.42 ± 11.63	28.03 ± 10.36	0.233
Proximal cap side-branch, *n*%	324 (79.02)	355 (73.65)	120 (74.53)	14 (60.87)	0.094
“Interventional” collaterals, *n*%	298 (72.68)	339 (70.33)	110 (68.32)	15 (65.22)	0.005
Diseased distal landing zone, *n*%	195 (47.56)	250 (51.86)	81 (50.31)	9 (39.13)	0.439
Contrast volume, ml	353.39 ± 157.71	372.50 ± 252.11	364.91 ± 257.59	342.17 ± 91.60	0.592
Procedural time, min	119.40 ± 68.41	115.62 ± 67.17	120.24 ± 67.17	148.65 ± 80.58	0.150
Procedural success, *n*%	377 (91.95)	441 (91.49)	143 (88.82)	21 (91.30)	0.687

eGFR, estimated glomerular filtration rate; LM, left main coronary artery; LAD, left anterior descending coronary artery; LCX, left circumtrunnion coronary artery; RCA, right coronary artery; CTO, chronic total occlusion; J-CTO, multicenter CTO registry in Japan; SYNTAX, Synergy Between PCI With Taxus and Cardiac Surgery.

Intraprocedural and in-hospital complications are shown in Table 3. There was no significant difference in the occurrence of intraprocedural complications and in-hospital MACE with decreased eGFR (*p* > 0.05). However, the incidence of other in-hospital complications, including pericardiocentesis, major bleeding, acute renal failure, and subcutaneous hematoma, were significantly higher (*p* < 0.05). Moreover, in the univariable and multivariable analysis of the CTO patients with low eGFR (<90 mL/min/1.73 m^2^) after successful revascularization, peripheral artery disease (OR: 5.778, 95% CI: 1.922–17.372, *p* = 0.002), lower platelet (OR: 0.993, 95% CI: 0.988–0.997, *p* = 0.002) and hemoglobin (OR: 0.982, 95% CI: 0.969–0.994, *p* = 0.003), higher uric acid (OR: 1.003, 95% CI: 1.001–1.005, *p* = 0.013), and calcification (OR: 1.852, 95% CI: 1.104–3.109, *p* = 0.020) independently increased the risk of in-hospital complications (Table 4).

**Table 3:** Intraprocedural and in-hospital complications.

	**eGFR ≥90** **mL/min/1.73 m^2^**	**90> e GFR ≥60** **mL/min/1.73 m^2^**	**60 > eGFR ≥30** **mL/min/1.73 m^2^**	**eGFR < 30** **mL/min/1.73 m^2^**	***p*-value**
	**(*n* = 410)**	**(*n* = 482)**	**(*n* = 161)**	**(*n* = 23)**	
Intraprocedural complications					
All-cause mortality, *n*%	2 (0.49)	0 (0)	0 (0)	0 (0)	0.354
Cardiac mortality, *n*%	2 (0.49)	0 (0)	0 (0)	0 (0)	0.354
Cardiac arrest, *n*%	1 (0.24)	0 (0)	0 (0)	0 (0)	0.654
Malignant arrhythmia, *n*%	0 (0)	0 (0)	0 (0)	0 (0)	–
Pericardial tamponade, *n*%	1 (0.24)	1 (0.21)	0 (0)	0 (0)	0.934
Pericardiocentesis, *n*%	1 (0.24)	0 (0)	0 (0)	0 (0)	0.654
Stroke, *n*%	0 (0)	0 (0)	0 (0)	0 (0)	–
Vascular dissection, *n*%	8 (1.95)	12 (2.49)	2 (1.24)	1 (4.35)	0.680
Collateral vessel perforation, *n*%	3 (0.73)	3 (0.62)	0 (0)	0 (0)	0.731
Loss of side branch ≥2 mm, *n*%	6 (1.46)	4 (0.83)	0 (0)	0 (0)	0.384
Acute renal failure, *n*%	0 (0)	0 (0)	0 (0)	0 (0)	–
Contrast allergy-related shock, *n*%	0 (0)	0 (0)	0 (0)	0 (0)	–
Vasovagal response, *n*%	0 (0)	1 (0.21)	0 (0)	0 (0)	0.745
In-hospital complications					
MACE, *n*%	17 (4.15)	21 (4.37)	10 (6.21)	1 (4.3)	0.748
All-cause mortality, *n*%	3 (0.73)	1 (0.21)	3 (1.86)	0 (0)	0.150
Cardiac mortality, *n*%	3 (0.73)	1 (0.21)	3 (1.86)	0 (0)	0.150
Non-fatal MI, *n*%	5 (1.22)	7 (1.45)	4 (2.48)	1 (4.35)	0.499
Clinically driven revascularization, *n*%	9 (2.20)	13 (2.70)	4 (2.48)	1 (4.35)	0.907
Emergency PCI	9 (2.20)	13 (2.70)	4 (2.48)	1 (4.35)	0.907
Emergency CABG	0 (0)	0 (0)	0 (0)	0 (0)	-
Other complications	24 (5.85)	49 (10.2)	25 (15.53)	7 (30.43)	< 0.001
Pericardiocentesis, *n*%	0 (0)	3 (0.62)	1 (0.62)	2 (8.70)	< 0.001
Cardiac arrest, *n*%	1 (0.24)	5 (1.04)	2 (1.24)	0 (0)	0.446
Malignant arrhythmia, *n*%	1 (0.24)	7 (1.45)	2 (1.24)	0 (0)	0.271
Cardiac shock, *n*%	3 (0.73)	5 (1.04)	1 (0.62)	1 (4.35)	0.345
Major bleeding, *n*%	17 (4.15)	21 (4.36)	18 (11.18)	6 (26.09)	< 0.001
Allergy-related shock, *n*%	1 (0.24)	0 (0)	1 (0.62)	0 (0)	0.447
Acute renal failure, *n*%	0 (0)	1 (0.21)	2 (1.24)	2 (8.70)	< 0.001
Subcutaneous hematoma, *n*%	6 (1.46)	22 (4.56)	8 (4.97)	1 (4.35)	0.049

eGFR, estimated glomerular filtration rate; MACE, major adverse cardiac event; MI, myocardial infarction; PCI, percutaneous coronary intervention; CABG, coronary artery bypass grafting.

**Table 4:** Univariable and multivariable logistic regression for in-hospital complications in patients with low eGFR (<90 mL/min/1.73 m^2^) after successful CTO-PCI.

	**Univariable analysis**			**Multivariable analysis**		
	**OR**	**95%CI**	***p*-Vvlue**	**OR**	**95%CI**	***p*-value**
Peripheral artery disease	5.951	2.350–15.066	< 0.001	5.778	1.922–17.372	0.002
Previous MI	1.702	1.098–2.638	0.017			
Platelet	0.994	0.990–0.998	0.003	0.993	0.988–0.997	0.002
Hemoglobin	0.979	0.968–0.989	< 0.001	0.982	0.969–0.994	0.003
CrCL	0.982	0.972–0.992	< 0.001			
eGFR	0.980	0.969–0.992	0.001			
Uric acid	1.004	1.002–1.006	< 0.001	1.003	1.001–1.005	0.013
Multivessel disease	2.936	1.157–7.450	0.023			
Multi-CTO lesion	1.708	1.094–2.669	0.019			
Calcification	2.326	1.495–3.618	< 0.001	1.852	1.104–3.109	0.020
J-CTO score	1.378	1.124–1.690	0.002			

eGFR, estimated glomerular filtration rate; CTO, chronic total occlusion; PCI, percutaneous coronary intervention; MI, myocardial infarction; CrCL, creatinine clearance; J-CTO, multicenter CTO registry in Japan; OR, odds ratio; CI, confidential interval.

Successful CTO-PCI significantly alleviated symptoms of dyspnea and angina in almost all patients at 1 month and 1 year, except those with eGFR<30 mL/min/1.73 m^2^ (Tables 5A,B). Notably, at 1 month, RDS scores had significantly decreased and SAQ-AS and SAQ-AF scores increased for all patients after successful CTO-PCI regardless of decreased eGFR (*p* < 0.01). Interestingly, changes to the RDS and SAQ scores were similar among all four groups (*p* > 0.05), indicating that successful CTO-PCI significantly alleviated symptoms of all CTO patients. At 1 year, RDS scores had remarkably decreased and SAQ-AS and SAQ-AF scores increased (*p* < 0.01) among patients with eGFR ≥30 mL/min/1.73 m^2^, while there were no significant changes in RDS and SAQ scores among those with eGFR < 30 mL/min/1.73 m^2^ (*p* < 0.01), demonstrating remarkable relief of symptoms for patients with low eGFR at 1 year after successful CTO-PCI, except those with eGFR <30 mL/min/1.73 m^2^. Additionally, after univariable and multivariable analysis, we found multi-CTO lesion (OR: 1.558, 95% CI: 1.077–2.254, *p* = 0.019) and tortuosity ≥45° (OR: 1.701, 95% CI: 1.182–2.449, *p* = 0.004) were independent risk factors for 1-year angina improvement in patients with low eGFR (<90 mL/min/1.73 m^2^) after successful CTO-PCI (Table 6).

**Table 5A:** Comparison of changes in RDS from baseline to follow-up.

		**RDS**
eGFR ≥90 mL/min/1.73 m^2^	Baseline	1.08 ± 1.08
	1 month follow-up	0.24 ± 0.54
	1 year follow-up	0.47 ± 0.68
	^m^Δ	0.86 ± 1.18
	^y^Δ	0.69 ± 1.25
	^m^*p*-value	< 0.001
	^y^*p*-value	< 0.001
90 > eGFR ≥ 60 mL/min/1.73 m^2^	Baseline	1.03 ± 1.12
	1 month follow-up	0.20 ± 0.49
	1 year follow-up	0.48 ± 0.66
	^m^Δ	0.84 ± 1.18
	^y^Δ	0.61 ± 1.23
	^m^*p*-value	< 0.001
	^y^*p*-value	< 0.001
60 > eGFR ≥ 30 mL/min/1.73 m^2^	Baseline	0.98 ± 1.06
	1 month follow-up	0.28 ± 0.61
	1 year follow-up	0.54 ± 0.66
	^m^Δ	0.71 ± 1.23
	^y^Δ	0.50 ± 1.16
	^m^*p*-value	< 0.001
	^y^*p*-value	< 0.001
eGFR < 30 mL/min/1.73 m^2^	Baseline	1.05 ± 1.07
	1 month follow-up	0.17 ± 0.51
	1 year follow-up	0.58 ± 0.79
	^m^Δ	0.90 ± 1.04
	^y^Δ	0.71 ± 1.27
	^m^*p*-value	0.003
	^y^*p*-value	0.201
	^a^*p*-value	0.518
	^b^*p*-value	0.215

RDS, rose dyspnea scale; eGFR, estimated glomerular filtration rate.

^*m*^Δ: change between baseline and 1 month follow-up.

^*y*^Δ: change between baseline and 1 year follow-up.

^*m*^p-value: baseline vs. 1 month follow-up.

^*y*^p-value: baseline vs. 1 year follow-up.

^*a*^p-value: comparison of ^*m*^Δ in the four groups.

^*b*^p-value: comparison of ^*y*^Δ in the four groups.

**Table 5B:** Comparison of changes in SAQ subscales from baseline to follow-up.

		**SAQ-PL**	**SAQ-AS**	**SAQ-AF**	**SAQ-TS**	**SAQ-DP**
eGFR ≥90 mL/min/1.73 m^2^	Baseline	63.14 ± 14.18	50.27 ± 15.36	78.51 ± 24.84	81.25 ± 14.82	72.28 ± 17.79
	1 month follow-up	68.25 ± 13.13	71.41 ± 23.83	97.86 ± 83.37	84.09 ± 11.73	72.14 ± 15.05
	1 year follow-up	68.04 ± 12.17	74.44 ± 25.90	95.08 ± 9.88	85.87 ± 9.92	78.06 ± 15.57
	^m^Δ	4.96 ± 19.58	21.34 ± 27.95	19.89 ± 25.77	2.54 ± 16.62	0.21 ± 21.68
	^y^Δ	5.58 ± 17.56	24.60 ± 29.72	18.15 ± 26.34	3.82 ± 17.69	5.96 ± 24.63
	^m^*p*-value	< 0.001	< 0.001	< 0.001	0.008	0.831
	^y^*p*-value	< 0.001	< 0.001	< 0.001	< 0.001	< 0.001
90 > eGFR ≥ 60 mL/min/1.73 m^2^	Baseline	63.97 ± 14.99	50.11 ± 16.94	78.66 ± 24.49	79.23 ± 15.11	69.27 ± 19.34
	1 month follow-up	69.09 ± 13.52	71.23 ± 23.95	98.16 ± 6.90	83.48 ± 11.19	72.78 ± 13.13
	1 year follow-up	67.58 ± 12.29	71.61 ± 26.83	94.53 ± 10.36	87.07 ± 10.24	79.17 ± 15.63
	^m^Δ	4.91 ± 19.14	21.23 ± 29.53	19.79 ± 24.70	4.18 ± 16.90	3.44 ± 22.19
	^y^Δ	3.97 ± 18.09	21.42 ± 32.50	17.24 ± 26.46	7.63 ± 19.22	10.00 ± 25.97
	^m^*p*-value	< 0.001	< 0.001	< 0.001	< 0.001	0.005
	^y^*p*-value	< 0.001	< 0.001	< 0.001	< 0.001	< 0.001
60 > eGFR ≥ 30 mL/min/1.73 m^2^	Baseline	59.30 ± 17.38	49.65 ± 18.53	74.55 ± 27.24	79.47 ± 15.64	66.20 ± 19.48
	1 month follow-up	67.30 ± 14.77	72.22 ± 24.37	98.30 ± 7.68	83.49 ± 11.21	69.14 ± 13.28
	1 year follow-up	65.95 ± 14.25	64.06 ± 32.57	91.88 ± 16.59	88.37 ± 10.35	78.58 ± 16.72
	^m^Δ	7.42 ± 20.41	22.59 ± 32.88	24.15 ± 27.44	3.83 ± 18.20	3.46 ± 22.01
	^y^Δ	6.42 ± 20.19	14.45 ± 35.72	18.83 ± 32.55	8.87 ± 19.22	13.48 ± 26.77
	^m^*p*-value	< 0.001	< 0.001	< 0.001	0.015	0.145
	^y^*p*-value	0.001	< 0.001	< 0.001	< 0.001	< 0.001
eGFR < 30 mL/min/1.73 m^2^	Baseline	59.15 ± 15.72	52.38 ± 10.91	82.86 ± 21.25	79.27 ± 10.94	66.27 ± 17.38
	1 month follow-up	68.64 ± 11.83	75.00 ± 24.25	97.22 ± 6.69	83.33 ± 11.46	68.98 ± 13.35
	1 year follow-up	61.48 ± 18.82	58.33 ± 35.89	92.50 ± 12.88	85.29 ± 8.87	78.47 ± 15.27
	^m^Δ	11.23 ± 18.27	23.61 ± 24.96	17.22 ± 21.09	3.27 ± 13.72	4.63 ± 19.85
	^y^Δ	3.89 ± 14.44	8.33 ± 38.92	6.67 ± 16.70	4.90 ± 14.15	13.89 ± 16.02
	^m^*p*-value	0.043	< 0.001	0.009	0.265	0.593
	^y^*p*-value	0.706	0.482	0.164	0.115	0.052
	^a^*p*-value	0.318	0.955	0.309	0.597	0.177
	^b^*p*-value	0.499	0.012	0.500	0.019	0.025

SAQ, seattle angina questionnaire; SAQ-PL, seattle angina questionnaire-physical limitation; SAQ-AS, seattle angina questionnaire-anginal stability; SAQ-AF, seattle angina questionnaire-anginal frequency; SAQ-TS, seattle angina questionnaire-treatment satisfaction; SAQ-DP, seattle angina questionnaire-disease perception; eGFR, estimated glomerular filtration rate.

^*m*^Δ: change between baseline and 1 month follow-up.

^*y*^Δ: change between baseline and 1 year follow-up.

^*m*^p-value: baseline vs. 1 month follow-up.

^*y*^p-value: baseline vs. 1 year follow-up.

^*a*^p-value: comparison of ^*m*^Δ in the four groups.

^*b*^p-value: comparison of ^*y*^Δ in the four groups.

**Table 6:** Univariable and multivariable logistic regression for 1-year angina improvement in patients with low eGFR (<90 mL/min/1.73 m^2^) after successful CTO-PCI.

	**Univariable analysis**			**Multivariable analysis**		
	**OR**	**95%CI**	***p*-value**	**OR**	**95%CI**	***p*-value**
Multi-CTO lesion	1.451	1.016–2.073	0.041	1.558	1.077–2.254	0.019
Lesion length	1.012	1.002–1.023	0.015			
Tortuosity ≥ 45°	1.711	1.195–2.449	0.003	1.701	1.182–2.449	0.004

eGFR, estimated glomerular filtration rate; CTO, chronic total occlusion; PCI, percutaneous coronary intervention; OR, odds ratio; CI, confidential interval.

Most importantly, this prospective study is the first to use the EQ-5D questionnaire to assess QOL of CTO patients with low eGFR. Surprisingly, at 1 month and 1 year after successful CTO-PCI, the EQ-5D scores of all patients had remarkably increased (*p* < 0.05) with similar improvement achieved by all four groups (*p* > 0.05, Table 7). Moreover, age (OR: 1.021, 95% CI: 1.003–1.039, *p* = 0.019) was an independent risk factor for 1-year QOL improvement in CTO patients with low eGFR (<90 mL/min/1.73 m^2^) after successful PCI (Table 8).

**Table 7:** Changes of EQ-5D in patients with successful CTO-PCI.

		**EQ-5D**
eGFR ≥90 mL/min/1.73 m^2^	Baseline	0.89 ± 0.17
	1 month follow-up	0.96 ± 0.11
	1 year follow-up	0.96 ± 0.11
	^m^Δ	0.07 ± 0.19
	^y^Δ	0.07 ± 0.20
	^m^*p*-value	< 0.001
	^y^*p*-value	< 0.001
90 > eGFR ≥ 60 mL/min/1.73 m^2^	Baseline	0.89 ± 0.16
	1 month follow-up	0.97 ± 0.09
	1 year follow-up	0.96 ± 0.10
	^m^Δ	0.07 ± 0.17
	^y^Δ	0.06 ± 0.18
	^m^*p*-value	< 0.001
	^y^*p*-value	< 0.001
60 > eGFR ≥ 30 mL/min/1.73 m^2^	Baseline	0.87 ± 0.18
	1 month follow-up	0.95 ± 0.12
	1 year follow-up	0.95 ± 0.11
	^m^Δ	0.08 ± 0.21
	^y^Δ	0.08 ± 0.22
	^m^*p*-value	< 0.001
	^y^*p*-value	< 0.001
eGFR < 30 mL/min/1.73 m^2^	Baseline	0.80 ± 0.23
	1 month follow-up	0.98 ± 0.08
	1 year follow-up	0.95 ± 0.11
	^m^Δ	0.19 ± 0.24
	^y^Δ	0.13 ± 0.29
	^m^*p*-value	0.003
	^y^*p*-value	0.036
	^a^*p*-value	0.052
	^b^*p*-value	0.640

CTO, chronic total occlusion; PCI, percutaneous coronary intervention; EQ-5D, European Quality of Life-5 Dimensions; eGFR, estimated glomerular filtration rate.

^*m*^Δ: change between baseline and 1 month follow-up.

^*y*^Δ: change between baseline and 1 year follow-up.

^*m*^p-value: baseline vs. 1 month follow-up.

^*y*^p-value: baseline vs. 1 year follow-up.

^*a*^p-value: comparison of ^*m*^Δ in the four groups.

^*b*^p-value: comparison of ^*y*^Δ in the four groups.

**Table 8:** Univariable logistic regression for 1-year QOL improvement in patients with low eGFR (<90 mL/min/1.73 m^2^) after successful CTO-PCI.

	**OR**	**95%CI**	***p*-value**
Age	1.021	1.003–1.039	0.019

eGFR, estimated glomerular filtration rate; CTO, chronic total occlusion; PCI, percutaneous coronary intervention; OR, odds ratio; CI, confidential interval.

These data demonstrate that successful CTO-PCI remarkably improved QOL of patients regardless of decreased eGFR and the degree of improvement was similar for patients with low and normal eGFR.

## Discussion

This is the first prospective study to comprehensively evaluate the effect of successful CTO-PCI on QOL of CTO patients with low eGFR. The main findings were as follows: (1) CTO patients with decreased eGFR tended to have more hypertension, diabetes, hyperuricemia, and previous stroke, in addition to lower hemoglobin and LVEF; (2) the incidence of in-hospital MACE was not increased, while there were significant increases in the frequency of pericardiocentesis, major bleeding, acute renal failure, and subcutaneous hematoma with decreased eGFR; (3) successful CTO-PCI significantly alleviated symptoms of all patients with eGFR ≥30 mL/min/1.73 m^2^ at 1 month and 1 year, but only at 1 month among those with eGFR <30 mL/min/1.73 m^2^; and (4) successful CTO-PCI significantly improved QOL regardless of eGFR at 1 month and 1 year to a similar degree for patients with low and normal eGFR. The real world study demonstrated that successful CTO-PCI improved QOL of patients with low eGFR.

Notably, low eGFR (<90 mL/min/1.73 m^2^) is fairly common in CTO patients, accounting for ~44.70–72.07% (16, 17, 21). In our study, low eGFR presented in 61.90% of CTO patients, consistent with the results of prior studies. Numerous studies reported that CTO patients with low eGFR were associated with higher proportion of comorbidities, including hypertension, diabetes, dyslipidemia, and low LVEF (13, 15, 17), which may be led by that renal impairment is associated with retention of sodium and water, inflammation, oxidative stress, metabolic perturbations (especially the disorder of lipid and lipoprotein metabolism), myocardial damage, and coronary microcirculation disorders (30, 31). Therefore, low eGFR was limited to a subset of CTO patients at high risk for the increased incidence of MCAE (11, 13, 14, 16, 17). According to the data obtained in this study, CTO patients with decreased eGFR who tended to be older and with hypertension and diabetes were a greater risk for CVD. Additionally, CTO patients with low eGFR were at greater risk for poorer cardiac function, manifested by significantly higher NT-proBNP and lower LVEF, which may be the important reason for the poor prognosis of CTO patients with low eGFR (12, 14–17). Additionally, we found that low eGFR was correlated with fewer “interventional” collaterals while no other researches have reported the correlation between low eGFR and “interventional” collaterals as of today. Werner et al. speculated that there may be some possible clinical determinants which damage collaterals, including diabetes, left ventricular (LV) function, myocardial viability, and angiographic factors such as collateral anatomy and size (32). In our study, CTO patients with low eGFR were found with more diabetes and worse LV function (low LVEF), indicating that the fewer “interventional” collaterals in low eGFR patients may somehow associate with the increased diabetes and worse LV function. However, we still need to further identify whether low eGFR directly damages the “interventional” collaterals and its potential mechanism.

Regarding the procedural safety and effectiveness for CTO patients with low eGFR previous literature has not been thoroughly compared and described. In the present study, we found not only the procedural success rate was not markedly reduced, but the occurrences of intraprocedural complications and in-hospital MACE were not significantly increased for CTO patients with low eGFR, indicating that revascularization is both safe and feasible for these patients. However, the incidence of bleeding-related adverse events, including pericardiocentesis, major bleeding, and subcutaneous hematoma, were markedly higher for CTO patients with low eGFR, similar to the findings of previous studies (33). These higher incidences of bleeding in low eGFR patients may be caused by platelet dysfunction, an imbalance in mediators of normal endothelial function, co-morbidities, such as vascular disease, hypertension, and anemia, and medical interventions for treatment of such co-morbidities (34–37). Additionally, the incidence of acute renal failure was higher in CTO patients with low eGFR, especially those with eGFR <30 mL/min/1.73 m^2^, indicating that safe contrast limits and aggressive periprocedural hydration for the subset of patients should be strictly considered. These findings confirm that with adequate preprocedural assessment and critical post-procedural care, timely CTO-PCI for patients with low eGFR is both safe and feasible.

The Global Expert Consensus Document states that the principal indication of CTO-PCI is improving symptoms (18). Successful CTO-PCI can significantly improve symptoms of dyspnea and angina (38–40). Additionally, studies reported that low eGFR was usually associated with multiple bothersome symptoms, including dyspnea, fatigue, depression and anxiety, and low eGFR was common in symptomatic CTO patients (19, 41). However, the impact of successful CTO-PCI on symptoms of CTO patients with low eGFR remains unclear, as only one study to date has reported that successful CTO-PCI significantly relieved symptoms of angina and dyspnea for patients with low eGFR at 1 month and 1 year (21). Similarly, in the present study, we further validated that successful CTO-PCI significantly relieved symptoms of CTO patients with eGFR ≥30 mL/min/1.73 m^2^ at 1 month and 1 year. Notably, for CTO patients with eGFR<30 mL/min/1.73 m^2^, successful PCI improved symptoms at 1 month with no significant difference at 1 year, which was inconsistent with the study cited above. Therefore, future large-sample randomized controlled trials are warranted to further assess the impact of successful CTO-PCI on symptoms of CTO patients with low eGFR, especially those with eGFR <30 mL/min/1.73 m^2^, aiming to provide stronger evidences for clinical practices. Additionally, we found that multi-CTO lesion was an independent risk factor for 1-year angina improvement in patients with low eGFR (<90 mL/min/1.73 m^2^) after successful CTO-PCI, which may be caused by that myocardial viability of patients with multi-CTO lesion was worse and the improvement of regional myocardial function after revascularization was poorer than those with single-CTO lesion, thereby leading to the less improvement of 1-year angina.

QOL is well-recognized as a critical indicator to assess status and perceptions of both physical and mental health (42). Independent of preventing MACE and relieving symptoms, it is imperative to determine whether successful CTO-PCI improved QOL (43). The DECISION-CTO (Drug-Eluting Stent Implantation vs. Optimal Medical Treatment in Patients With Chronic Total Occlusion) trial (*n* = 417) reported that CTO-PCI significantly improved QOL, as determined with the EQ-5D questionnaire, at 1 month and these improvements were largely maintained at 12 and 36 months (23). The EUROCTO (Evaluate the Utilization of Revascularization or Optimal Medical Therapy for the Treatment of Chronic Total Coronary Occlusions) trial (n = 259), which also used the EQ-5D questionnaire, found that successful CTO-PCI greatly improved QOL of patients at 12 months (44). Renal impairment has an enormous impact on an individual's QOL and QOL is significantly reduced with the decrease of eGFR, which may be caused by increased suffering from chronic illness (e.g., hypertension, diabetes, congestive heart failure, and cancer) and multiple physical and psychological symptoms (e.g., pain, dyspnea, fatigue, sleep disturbances, nausea and vomiting, cognitive impairment, and anxiety and depression) (20, 41, 45). However, the two RCT did not specify the QOL in the CTO patients with low eGFR, and to our knowledge, our study is the first prospective clinical trial to investigate the impact of successful CTO-PCI on QOL of CTO patients with low eGFR. To address this critical knowledge gap in clinical practice, we used the EQ-5D questionnaire and found that successful CTO-PCI remarkably improved QOL of CTO patients with low and normal eGFR at 1 month and 1 year.

## Study limitations

There were some limitations to this study that should be addressed. First, the single-team nature of this study is a potential weakness that may be not suitable for other centers and teams. Second, the number of CTO patients with eGFR <30 mL/min/1.73 m^2^ was relatively small with significant differences as compared to other groups and may have resulted in statistical bias, which is difficult to avoid in a real-world study. Third, this study did not include patients who were either not provided PCI or referred for surgical revascularization. Finally, objective measurements of physical capacities, such as those from exercise stress testing, were not systematically conducted during the follow-up.

## Conclusion

The results of the present study demonstrated that timely and successful CTO-PCI achieved substantial symptom relief and improved QOL of patients with low eGFR, although the risks of MACE and all-cause mortality were not effectively reduced.

## Data availability statement

The raw data supporting the conclusions of this article will be made available by the authors, without undue reservation.

## Ethics statement

The studies involving human participants were reviewed and approved by Ethics Committee of Xijing Hospital (Approval No. KY20172019-1). The patients/participants provided their written informed consent to participate in this study.

## Author contributions

SZ, YC, BZ, KL, CL, and HG were involved in the study design and drafted the manuscript. JW, ZW, YZ, WH, GC, TY, and PH collected data and performed the follow-up for this study. SZ, BZ, WW, and ZZ conducted statistical analysis. HW, CX, and LY researched data and contributed to discussion. All authors read and approved the final manuscript.
